# Theoretical concepts and instruments for measuring hospital discharge readiness: A scoping review

**DOI:** 10.1016/j.heliyon.2024.e26554

**Published:** 2024-02-21

**Authors:** Hanna Feldbusch, Marita Schmidt, Eva Maria Steeb, Natalie Paschek, Maren Nemesch, Yannick Sartory, Rebekka Brenner, Stefan Nöst

**Affiliations:** a Baden-Württemberg Cooperative State University Stuttgart, School of Health Sciences and Management, Stuttgart, Germany; b Robert Bosch Hospital, Stuttgart, Germany

**Keywords:** Discharge readiness, Hospitals, Inpatients, Nursing, Patient discharge, Health care surveys, Continuity of patient care, Scoping review

## Abstract

**Background:**

The Discharge Readiness of adult patients in a hospital setting is a multidimensional concept which is becoming increasingly important internationally as part of discharge planning. To date, there has been a lack of reviews of existing measurement instruments as well as theoretical concepts of discharge readiness.

**Objective:**

To provide an overview of existing measurement instruments and theoretical concepts regarding readiness for hospital discharge in adult patients.

**Design and methods:**

A scoping review was conducted in accordance with the Joanna Briggs Institute methodological manual and PRISMA ScR reporting principles. A literature search was conducted using the CINAHL and LIVIVO databases (including MEDLINE and PSYINDEX) in October 2021. After test screening, all identified articles were screened by two independent reviewers using predefined inclusion and exclusion criteria before the content was extracted and mapped.

**Results:**

Of the 1823 records identified, 107 were included in this review. Of these, 30 studies were included as development or validation studies of measurement instruments assessing discharge readiness, 68 were included as empirical studies with readiness for hospital discharge as the primary outcome or key concept, and nine publications were included as theoretical papers or reviews. Five dimensions of readiness for hospital discharge were extracted:1) Physical, 2) Psychological, 3) Education and Knowledge, 4) Adequate Individual Support, and 5) Social and Organisational Determinants. Of the 47 instruments identified for measuring discharge readiness, 33 were validated. The Readiness for Hospital Discharge Scale (RHDS) was the most frequently used instrument.

**Conclusions:**

The systematic measurement of readiness for hospital discharge, particularly from the patient's perspective combined with the nurse's perspective, might be useful in reducing negative outcomes such as readmissions. This review provides an overview of existing and validated instruments for the systematic assessment of discharge readiness in acute inpatient care, as well as an overview of the theoretical concepts of readiness for hospital discharge. Further research is needed on the relationship between organisational determinants and readiness for discharge.

## What is already known

• Comprehensive discharge planning increases patient safety and satisfaction and leads to improved continuity and quality of care.
• Assessing readiness for hospital discharge can reveal unmet patient needs related to transition and continued care, thereby actively promoting high-quality discharge planning.
• There are no identified internationally standardised definitions of readiness for hospital discharge, nor systematic reviews of assessment tools.

## What this paper adds

• Various theoretical concepts of hospital discharge readiness have been described in the literature and are now systematically displayed for further research and practice development.
• Despite instruments existing to measure hospital discharge readiness, there is a still need for consensus on the definition and measurement of as well as more on factors that influence hospital discharge readiness.

## 1 Background

Adequate discharge planning is becoming increasingly important in the context of increased multimorbidity and chronic diseases, especially among older people [1,2]. Discharge planning is an interdisciplinary intervention designed to ensure a successful transition from hospital to post-hospital care, thus promoting the continuity of care. Discharge planning can facilitate successful transitions, promote recovery, and lead to better care outcomes such as lower readmission and complication rates, higher patient satisfaction, and better health [[3], [4], [5]]. Meleis's middle-range theory of transitions fundamentally covers the characteristics of transitions and people's experiences of transition, which are multidimensional. This theory consists of the types and patterns of transitions, properties of transition experiences, facilitating and inhibiting conditions, process indicators, outcome indicators, and nursing therapeutics. Anticipatory preparation can facilitate the transition experience, whereas a lack of preparation is an inhibitor [6]. Following Titler and Pettit, readiness for discharge can be considered part of discharge planning [7]. Discharge readiness refers to the perception or judgement regarding the preparation or lack of it for hospital discharge [8,9]. It is both a state as well as a process [10]. Discharge readiness was first used in a psychiatric setting in 1995 by Titler and Pettit as a multidimensional construct to assess patients' and family members' abilities to leave an acute care facility [7].

The measurement of discharge readiness using survey instruments has been established in some countries such as the USA, Turkey, Indonesia, and China [[11], [12], [13], [14]]. In Germany, the concept of discharge readiness is gaining importance, although no validated assessment instrument is available in the German language [15]. Since 2019, the German national expert standard, “Discharge Management in Nursing” recommends the assessment of discharge readiness as part of hospital discharge management and points out that discharge readiness is an indicator of the outcome quality of discharge planning [16]. The expert standards of the German Network for Quality Development in Nursing (DNQP) are instruments used for nursing-related quality promotion and assurance representing a quality standard. For continuous quality improvement, an expert standard defines the quality objectives and assessment criteria for nursing-related topics.

Weiss and Piacentine made relevant contributions to research on the concept of readiness for hospital discharge and its measurement [11]. Among others, they considered the importance of assessing patients’ readiness for discharge as an outcome measure for hospitalisation and a predictor of post-discharge outcomes. Despite the growing international importance of the concept and measurement of readiness for hospital discharge, with the exception of a concept analysis by Galvin et al. [10], no review of the theoretical concepts or dimensions of readiness for discharge and, in particular, no review of existing survey instruments could be identified.

Theoretically, a positive correlation between discharge planning and transition is assumed. In order to measure the quality of discharge planning in nursing practice and to enable improvements, discharge readiness can be formulated as an indicator of the quality of discharge planning. Based on this consideration, the measurement of discharge readiness is becoming increasingly important in daily nursing practice; however, the research situation remains confusing. This review aims to provide an overview of the existing theoretical concepts of hospital discharge readiness and instruments for systematic measurement. The primary and secondary research questions are summarized in Table 1.

**Table 1:** Core contents of the literature review protocol.

Primary research question	1) What theoretical concepts on hospital discharge readiness are reported? 2) What instruments for the systematic assessment of readiness for hospital discharge are available?			
**Secondary research questions**	To 1) and 2)	• For what population is the identified theoretical concept/assessment tool designed? • For what geographical and clinical care context was the concept/assessment tool developed or applied?		
	To 1)	• What outcomes can be used to check the readiness of patients to be discharged? • What benefits and limitations of the concept are described?		
	To 2)	• What self- or third-party reported assessment instruments of readiness for hospital discharge are reported? • What is the theoretical foundation of the readiness for hospital discharge assessment instrument? • For what purpose is the assessment instrument intended? • What evidence is available on the goodness of fit of the assessment instruments? • Are evaluation studies available for the identified assessment instruments?		
**Review Objectives**	• Overview of existing theoretical concepts and assessment tools of readiness for hospital discharge in the clinical care context:			
	Identification of different dimensions of discharge readiness and to elicit the operationalization of discharge readiness			
	• Mapping of concepts and research approaches on the topic of discharge readiness in hospitals			
**Search strategy**	Inclusion criteria	Population	Adults	
		Setting	Hospital; acute inpatient care setting	
		Language	German; English	
		Instrument/concept	E1	Development/validation of tools to measure readiness for hospital discharge, including translations and cultural adaptions of validated measurement tools of readiness for hospital discharge or preparedness as predictor of an outcome on patient level.
			E2	Empirical study formulating readiness for hospital discharge as a primary outcome (quantitative) or identifying discharge readiness as a key concept or category (qualitative) - (not primarily for development/validation). If empirical studies match the inclusion criteria of E1, they are labelled as E1, not E2.
			E3	Theoretical papers developing or discussing operational concepts of discharge readiness, including reviews.
	Exclusion criteria	Population	Children; parents; relatives of patients; other stakeholders who are not part of the adult patient population.	
		Setting	Outpatient care settings; long-term care settings; other care settings (except acute inpatient care)	
		Language	Other languages besides German and English	
		Instrument/concept	Articles that do not have discharge management or discharge process or readiness for hospital discharge as a research topic; readiness for hospital discharge only as a secondary outcome in intervention studies or subcategory or secondary concept in qualitative studies.	
**Data sources**	Bibliographic searches	CINAHL Complete; LIVIVO (included databases: BASE, Catalogue of the NML, Catalogue ZB MED, Current Contents, DissOnline, ETHMED, EZB, HECLINET, Medline, Publishing data, SOMED, PSYINDEX)		
	Grey Literature	Dissertations		
	Manual Search	Reference lists		
**Search terms in electronic databases**	CINAHL Complete	[Open Search]: (Readiness OR Entlassbereitschaft OR Entlassungsbereitschaft OR Entlassfähigkeit OR Entlassungsfähigkeit) AND (Hospital OR Krankenhaus OR Klinik) AND (Discharge OR Entlassung OR Entlassungsmanagement OR Entlassmanagement)		
	LIVIVO	[TI Title] OR [AB Abstract]: (Readiness OR Preparedness OR Entlassbereitschaft OR Entlassungsbereitschaft OR Entlassfähigkeit OR Entlassungsfähigkeit) AND (Hospital OR Krankenhaus OR Klinik) AND (Discharge OR Entlassung OR Entlassmanagement OR Entlassungsmanagement)		

## 2 Methods

A scoping review was conducted to provide an overview of existing theoretical concepts and assessment instruments for readiness for hospital discharge according to the Joanna Briggs Institute methodological manual and PRISMA ScR reporting principles [17,18].

### 2.1 Scoping review protocol

Prior to performing the Scoping Review, a Scoping Review Protocol was developed from August to October 2021, based on the framework of Arksey and O'Malley and the Joanna Briggs Institute methodological manual [18,19]. A structure for the successful implementation of this study was developed, including the study objectives, research questions, inclusion and exclusion criteria, search strategies, and data sources. Table 1 lists the core contents of the protocol.

### 2.2 Search strategy

A literature search was conducted to obtain a broad overview of readiness and preparedness for hospital discharge. A literature search of the electronic databases CINAHL and LIVIVO, including MEDLINE, was performed in October 2021 (Table 1). Parallel to the data extraction, additional validation studies of the already identified assessment instruments were manually searched via reference lists.

### 2.3 Literature screening

The inclusion and exclusion criteria formulated in the study protocol were tested for applicability using a test screening conducted in October 2021 on a sample of 76 identified articles. Screening was divided into two phases. First, articles were excluded and preselected based on titles and abstracts using the Rayyan software [20]. Articles that could not be excluded with certainty were screened based on their full text. In the second phase, the remaining articles were included or excluded based on their full texts. Two independent reviewers reviewed the articles during both phases of the screening process. A total of eight reviewers were involved in the test screening and screening process. In case of discrepancies in inclusion and exclusion, these were discussed by the reviewer team or project group after unblinding, and the results were recorded.

No exclusion was made based on the publication date. The articles included were divided into studies on development or validation of an instrument for measuring readiness for hospital discharge (e1), empirical studies with readiness for hospital discharge as the primary outcome or key concept (e2) and theoretical research, including reviews (e3). Empirical studies matching the inclusion criteria for instrument development or validation (e1) were classified as (e1) not (e2) (Table 1).

### 2.4 Extraction and charting the data

All included articles were saved in Citavi 6 software and subsequently analysed and extracted based on the research questions. These included source, study design, geographical context, target group and study population. Geographical context was defined as the country in which the study was conducted. If this was not mentioned, the authors’ affiliations were used. In case of two countries or more, “international” was used for documentation. Depending on the inclusion category (e1, e2, e3), variables such as data type, study objective, assessment or measurement instrument, assessment perspective, purpose of the measurement and definition or operationalization of readiness for hospital discharge were also extracted (additional file 1 Tables S1–S3). The papers included as theoretical concepts (e3) are mapped in the results section. Based on the heterogeneity of the empirical and validation or development studies, articles (e1, e2) using, developing, or validating a measurement instrument for the assessment of discharge readiness were mapped to the results. Studies using a qualitative research approach were not systematically mapped in the results section. The results of the data extraction of the included articles are reported in the additional file 1 Tables S1–S3.

## 3 Results

**Fig. 1:**
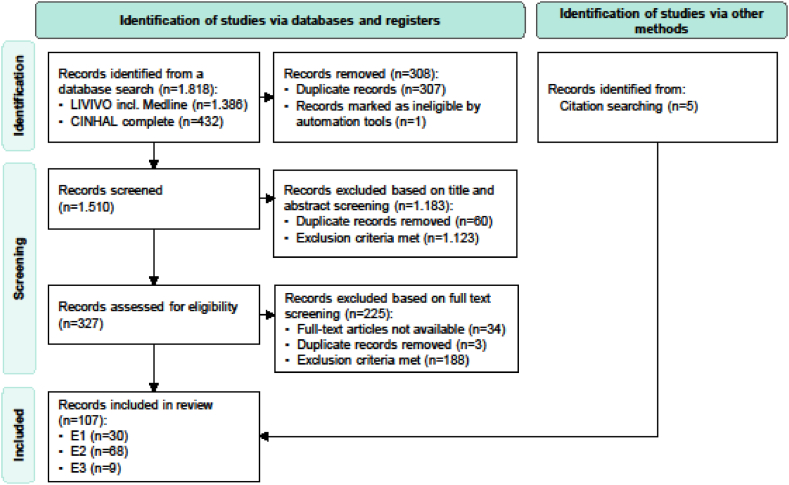
Flow diagram of the study selection. *Note:* E1 = Development or validation studies; E2 = Empirical studies with discharge readiness as the primary outcome or key concept; E3 = Theoretical papers and reviews.

The literature search yielded 1823 articles, and after screening, 107 publications were included in the review. Based on the included publications, 30 studies were included as validation or development studies of an instrument for measuring readiness for hospital discharge (e1), 68 as empirical studies with discharge readiness as the primary outcome or key concept (e2), and nine publications as theoretical papers, including reviews (e3). Fig. 1 shows a flowchart of the screening process according to the PRISMA ScR reporting principles [21]. The summary and list of all included studies with the corresponding inclusion categories are illustrated in additional file 1 Tables S1–S3.

### 3.1 Characteristics of selected articles

The included studies were generated from various countries: 42 publications from the United States, 13 from China, eight each from Canada and Australia, five from Switzerland and Poland, four studies undertaken in two or more countries, as well as four studies from Turkey, three studies each from Indonesia, Ireland, and Thailand, two publications each from South Africa and the UK and one study each from Belgium, Denmark, Iran, Japan, and the Netherlands.

Of the 30 validation or development studies on instruments used to measure discharge readiness (e1), 29 had a quantitative design. Of these, 23 were validation studies, five were observational studies and one was an experimental study. In one study, the design could not be evaluated [9]. Among the 68 empirical studies with readiness for hospital discharge as the primary outcome or key concept (e2), 60 had a quantitative design, of which three were RCTs, six were quasi-experimental studies, and 51 were observational studies. Five of the empirical studies had a qualitative design and three studies had a mixed-methods design. The nine theoretical papers, including reviews (e3), consisted of four systematic reviews [[22], [23], [24], [25]], two literature reviews [7,10], one discussion paper [26], one study with an underlying Delphi process [27], and one dissertation [28].

The studies addressed a wide range of target groups and study populations with different sample sizes. For example, in addition to surgical patients, people with psychiatric diagnoses, veterans, and people with HIV or cancer were examined. The smallest reported sample consisted of five patients [29], and the largest consisted of data from 18,203 adults [30]. For more detailed information on the geographical context, study design, and target group or study population, see additional file 1 Tables S1–S3.

### 3.2 Dimensions of readiness for hospital discharge

Five main dimensions or categories were identified from nine theoretical studies and reviews: physical dimension, psychological dimension, education and knowledge, adequate individual support, and social and organisational determinants. An overview of the definitions, operationalisations, and factors of readiness for hospital discharge provided in the single studies can be found in additional file 1 Table S1. Galvin et al., and Titler and Pettit reported on discharge readiness across patient groups, for which the term “generic” is used [7,10]. Of these, the study by Galvin et al. is a concept analysis in which the attributes, antecedents, and consequences of readiness for hospital discharge were identified, providing the basis for an operational definition and conceptual framework of discharge readiness [10]. The remaining seven studies reported discharge readiness using the following target groups: patients after total knee [26] or hip replacement [25], patients following colorectal surgery [24,27], older patients [22,23], and ante-, intra- and postpartum mothers [28].

#### 3.2.1 Physical dimension

The physical dimension describes the functional readiness for discharge from hospital. Titler and Pettit labelled this phenomenon as the physiological stability of the patient [7] and Galvin named it physical stability and described it in more detail with the following antecedents: stable vital signs, adequate intake and output, normal elimination, adequate ambulation, minimal bleeding, pain control, absence of nausea or vomiting, functional ability, and competence to manage self-care at home [10]. These antecedents match the themes, criteria, and influencing factors reported in other patient group-specific studies and can be attributed to physical preparedness. Fiore et al. listed tolerance or tolerability of oral intake, recovery of lower gastrointestinal or bowel function, ability to mobilise and self-care or adequate mobility, and, in addition, clinical examination and laboratory tests showed no evidence of complications or untreated medical problems for determining readiness for hospital discharge after colorectal surgery [24,27]. De Morton stated that independent mobility was a key factor in determining discharge readiness in a target group of older acute medical patients [23]. Finally, Supattra et al. reported physiological experiences as a major theme influencing the readiness of patients for hospital discharge after total hip replacement, subdividing them into recovery of mobility and physical safety [25].

#### 3.2.2 Psychological dimension

The psychological dimension is related to the competence and mental ability to leave the hospital. Supattra et al. described this as psychological experiences and coping ability [25]. Titler and Pettit related patients' and families’ cognitive and psychomotor competencies to carry out self-care management regimens and perceived self-efficacy to perform self-care management regimes, which are measured with a discharge readiness assessment [7]. Galvin et al. labelled this dimension of psychological ability and described confidence, competence, conception of life at home, coping, control, and empowerment as associated antecedents [10].

#### 3.2.3 Education and knowledge

Education and knowledge as a dimension of discharge readiness can be evaluated if the information, education, knowledge, and training received correspond to the post-hospital needs. Causey-Upton et al. cited education as an influencing factor in individuals after total knee replacement [26]. Likewise, Malagon-Maldonado described the delivery of education as a significant predictor of readiness for hospital discharge in mothers [28]. Supattra et al. outlined the need for information from patients after a total hip replacement during and after hospitalisation [25]. Galvin et al. also examined this topic under adequate information and knowledge, which is further described by the following characteristics: caring for oneself, personal needs, problems which may occur, whom and when to call for physical restrictions, what happens next, and available services [10].

#### 3.2.4 Adequate individual support

Adequate individual support can be interpreted as not only physical support but also emotional, psychological, and other support for a person to be able to transition into a post-hospital setting [28]. The influencing factors, themes, or criteria mentioned in the literature are availability of social support [7,26], family support after discharge, and needs from the healthcare team [25]. Furthermore, adequate analgesia or pain control can be considered adequate medical support, although some literature classifies this as physical readiness [10]. Galvin et al. characterised adequate support as antecedent emotional and physical support [10].

#### 3.2.5 Social and organisational determinants

Influencing factors, issues, and domains of readiness for hospital discharge at the individual or organisational level which could not be attributed to the physical and psychological dimensions or categories, such as education and knowledge or adequate individual support, were assigned to the dimension of social and organisational determinants.

At the individual level, patient characteristics, such as age, complications, and presurgical functional level, were included. For the target group of mothers, the length of stay of the infant was considered [26,28]. The four main characteristics associated with postpartum readiness mentioned by Malagon-Maldonado are maternal sociodemographic characteristics, prenatal characteristics such as prenatal class and care, perinatal factors (e.g. delivery method and timing, neonatal characteristics such as lower birth weight or neonatal medical problems in the hospital), and postpartum characteristics (e.g. feeding method) [28].

Considering the individual as well as organisational levels, individual access to the healthcare system and community resources should also be considered [7], along with the availability and quality of pre-rehabilitation and rehabilitation to improve readiness at discharge [26].

At the organisational level, provider characteristics such as practitioner age, number of years practising, practitioner certification, and educational topics covered in the hospital are associated with postpartum readiness [28]. Table 2 provides an overview of the identified dimensions and factors associated with them.

**Table 2:** Overview of identified dimensions and factors from the theoretical papers (e3) influencing readiness for hospital discharge.

Dimensions and specific factors influencing readiness for hospital discharge		Reported target Group	Sources
**Physical dimension**		Generic	[7,10]
	Physical safety	Patients after total hip replacement	[25]
	No indication of complications or untreated medical problems	Patients following colorectal surgery	[27]
	Recovery of lower gastrointestinal/bowel function	Patients following colorectal surgery	[24,27]
	Tolerance of oral intake	Patients following colorectal surgery	[24,27]
	(Recovering) mobility	Older patients and patients following colorectal surgery	[23,24,27]
	Ability/Competence for self-care	Generic and patients following colorectal surgery	[10,27]
**Psychological dimension**		Generic	[10,27]
	Competency and perceived self-efficacy to carry out self-care management regimens	Generic	[7,10]
	Psychological experiences	Patients after total hip replacement	[25]
	Coping ability	Patients after total hip replacement	[25]
**Education and knowledge**		Generic, mothers and patients after total knee replacement	[10,26,28]
	Information needs from the healthcare team	Patients after total hip replacement	[25]
**Adequate individual support**		Generic	[7,10]
	Social support	Generic and patients after total knee replacement	[7,26]
	Family support after discharge	Patients after total hip replacement	[25]
	Analgesia/adequate pain control with oral analgesia	Patients after total knee replacement and colorectal surgery	[26,27]
	Needs from the healthcare team	Patients after total hip replacement	[25]
**Social and organisational determinants**		–	–
	Patient characteristics	Mothers and patients after total knee replacement	[26,28]
	Infant length of stay	Mothers	[28]
	Access to the health care system and community resources	Generic	[7]
	Provider characteristics	Mothers	[28]
	Pre-rehabilitation and rehabilitation (setting)	Patients after total knee replacement	[26]

### 3.3 Instruments to assess readiness for hospital discharge

A total of 47 instruments were identified to measure readiness for hospital discharge, preparedness, or dimensions of discharge readiness. For these assessment instruments, the first step was to identify instrument development and validation studies. In addition, seven validation studies were identified through manual searches using reference lists and databases, and only five of these seven studies were written in German or English. Of the 47 instruments, two instruments were developmental studies without identifiable (German or English) validation studies [9,31]. As empirical evidence, twelve studies were classified in which instruments such as self-developed questionnaires [32,33] or single items [[34], [35], [36], [37]] were used to measure readiness for discharge, for which no German or English language validation study could be identified. One of the 33 validated instruments was a single item [38] and the other was a German translation of the Readiness for Hospital Discharge Scale/Short Form [39]; however, according to the authors, further studies on the concept of patient readiness to leave the hospital as well as assessing the validity and reliability of the questionnaire are needed before the instrument can be used systematically. Owing to these results, neither study was included in the systematic overview of the validated instruments for readiness to discharge measurement instruments in Table 3.

**Table 3:** Overview of validated instruments to measure patients’ readiness for hospital discharge.

Measurement tool		Purpose of the measurement	Assessment perspectives	Empirical studies (e2)	Development/validation studies (e1)
Readiness for Hospital Discharge Scale (**RHDS**)					

	RHDS (21 items) (Master version)	RHD	Self-reported	[40]	[11,41,42]
			Self-reported; physiotherapist-reported	[43]	
	(PT)-RHDS (22 or 23 items)		Self-reported	[3,4,44,[45], [46], [47], [48], [49], [50]]	
	(PT)-RHDS/SF (8 items)		Self-reported	[30,[51], [52], [53], [54], [55]]	[56,57,58]
	RHDS-OP (23 items)	Older adults' RHD			[59]
	RHDS-OP/SF (9 items)				
	RHD-MIS (23 items)	RHD after myocardial infarction		[60,61]	[62]
	RHDS-After Birth Scale (9 items)	Mothers RHD		[63]	
	RHDS-New Mother Form (22 items)			[64,65]	[66]
	RN-RHDS (21 or23 items)	RHD	Nurse-/(caregiver-) reported	[45,46]	[67,42]
	RN-RHDS/SF (8 items)			[30,51,53]	[56,58]
			Physician-reported	[51]	

Readiness for Hospital Discharge Scale (**RHDS**) – Translated					

	Bahasa-RHDS-Parents (22 items)	Parents RHD	Nurse-reported		[13]
	Polish version RHDS for postpartum mothers (23 items)	Postpartum women's RHD	Self-reported		[68]
	Turkish version of the RHDS-New Mother Form (23 items)			[69]	
	Turkish version of the RHDS/SF (8 items)	RHD		[70,71]	[12]
	RHDS-Fr (20 items)				[72]
	(Modified) Chinese Version of the RHDS (23 items)			[[73], [74], [75]]	[76]
	Chinese version of the RHDS (12 items)			[77]	
	Chinese version of the RHDS (22 items)			[[78], [79], [80], [81], [82], [83]]	[14]
	Indonesian version of the RHDS (20 or 29 items)			[84]	[85]
	Thai translation of the PT-RHDS (23 items)			[86]	[87]

Others					

The Discharge Readiness Inventory (**DRI**)		Discharge readiness	Third party-reported	[88,89,90]	[91]
**PREPARED** questionnaire (50 items)		Quality of discharge care planning/discharge preparation or preparedness	Self-reported	[92,93,93,94]	[95]
	B-PREPARED (11 items)	Discharge preparedness		[96]	[97,98]
The Care Transitions Measure (**CTM**) (15 items)		Discharge readiness/quality of transition	Self-reported	[99]	[100]
	Dutch translation of the CTM				
	CTM-3 (3 items)				[98]
Readiness for Discharge Questionnaire (**RDQ**) (6 items)		Readiness for discharge	Third party-reported		[101]
Perceived Readiness for Discharge after Birth Scale (**PRDBS**) (9 items)		New mothers RHD	Self-reported		[66]
**FIM**^**TM**^ (18 items)		Patients' functional status	Third party-reported		[102]
Modified Post Total Hip Replacement Discharge Scoring Scale (**PTHRDSS**) (9 items)		Home-readiness	Third party-reported		[103]
Discharge Planning Questionnaire (**DPQ**) (51 items)		Discharge needs	Self-reported	[104]	[105]

*Note:* RHD = Readiness for hospital discharge; PT = Adult medical-surgical form; SF = Short form; OP = Older people; MIS = Myocardial infarction; RN = Registered nurse; MD = Physician reported; Fr = French version.

The assessment instrument used in 37 empirical studies (e2) and 18 development or validation studies (e1), and thus the most frequently used or thematised identified instrument, are variations of the Readiness for Hospital Discharge Scale (RHDS). The second most-used assessment instrument in four empirical (e2) and three validation studies (e1) was the PREPARED or B-PREPARED questionnaire. The third most frequently used instrument in one development and validation study and in three empirical studies was the Discharge Readiness Inventory (DRI). The Care Transition Measure (CTM), Readiness for Discharge Questionnaire (RDQ), Modified Post-Total Hip Replacement Discharge Scoring Scale and Discharge Planning Questionnaire (DPQ) were each used in one to three identified studies. Furthermore, the Perceived Readiness for Discharge After Birth Scale provided a basis for the development of the RHDS [11,66].

#### 3.3.1 The readiness for hospital discharge scale

Weiss developed the RHDS to assess patient perceptions of their readiness for hospital discharge. The underlying construct of discharge readiness was specified as the judgement or perception of the patient's immediate state and perceived abilities related to managing care needs in the home environment. This questionnaire initially consisted of 23 items, and after evaluating the psychometric properties (item and scale characteristics, internal consistency reliability, construct validity, and predictive validity) of 21 items, it was developed for use across patient populations after the decision to discharge [11]. For the revised scale a Cronbach's alpha coefficient of 0.89 was reported for the overall scale and 0.74 to 0.90 for the subscales [11,41]. The 21 items of the scale were categorised into four subscales to measure the following four components: 1) patient's physical-emotional state before discharge (personal status), 2) perceived adequacy of information needed to respond to common concerns and problems after discharge (knowledge), 3) perceived ability to self-manage personal and healthcare needs post-discharge (coping ability), and 4) expected availability of emotional and instrumental assistance after discharge (expected support). Items are scored on an 11-point Likert scale (zero–ten). The higher the score, the greater the patient's perceived discharge readiness [11]. In some studies, such as those by Bobay et al. and Coffey et al., a cut-off value less than seven was used for low readiness [41,44]. The total and subscale scores were calculated by summing the numerical responses for each item.

Based on Weiss’ RHDS a total of 21 different RHDS scales were identified. These are short forms of the RHDS [56], scales for assessment from the perspective of other stakeholders such as nurses [56,67], translations, cultural adaptations [68], and scales (modified) for specific patient groups: older adults [72], adults after myocardial infarction [62], medical-surgical patients [44], and postpartum mothers [11].

#### 3.3.2 The PREPARED-questionnaire

The 50-item PREPARED questionnaire was developed by Grimmer and Moos in 1998 to assess the quality of discharge planning from the patient's perspective, and was validated for use with older adults [97,95]. Quality scores were calculated for four domains: 1) support structures and information exchange, 2) medication and management issues, 3) concerns with community management and preparedness to deal with unexpected issues, and 4) control of discharge circumstances. The questionnaire was structured into eight parts and addressed patients' perceptions of their experiences during hospitalisation (parts one to four) and after being discharged (parts five to eight). Each part contained 3 to 8 questions, including dichotomous yes/no closed-ended questions, open-ended questions, and questions with multiple levels that differed based on the degree of the presence or absence of the phenomenon [92]. The Brief-PREPARED is a validated short version of the PREPARED with eleven items. Based on studies by Blumer et al. and Graumlich et al., it was used and validated to measure patient preparedness for hospital discharge [97,96].

#### 3.3.3 The discharge readiness inventory

The DRI by Hogarty and Ulrich was primarily developed to assess the potential for discharging patients with long-term stays in psychiatric hospitals back into the community [88]. It provides further clinical rating scores for total discharge readiness and four subscale categories valued by a clinical personnel for psychiatric inpatients: 1) community adjustment potential (CAP) measuring patient's potential for adjustment in terms of ability to perform appropriate social behaviours after discharge in 16 items; 2) psychosocial adequacy (ADE) by assessing the individual's mental health including dimensions as self-care, personal hygiene, and instrumental role performance in 16 items; 3) belligerence (BEL) by measuring aggressiveness and hostility in six items; and 4) manifest psychopathology (MAN) by evaluating active and disruptive hallucinations or delusions in three items. The clinicians indicate their DRI ratings on a five-point scale, from “no problem” to “severe problem”, for each item. The total discharge readiness score can be determined using following formular: DRI = CAP + ADE − BEL − MAN [89]. The reliability and validity of the DRI were successfully evaluated [91].

## 4 Discussion

As one aim of this study was to provide an overview of existing theoretical concepts, this was realised by identifying the five dimensions of discharge readiness (Table 2). The five dimensions extracted from the theoretical research appear to be close to the theoretical foundation of the identified measurement instruments. For example, the RHDS includes four of the dimensions in its subscales [11]. The domains of the PREPARED questionnaire were also included in the dimensions except for medication issues, which were not specifically listed [92]. An exception was the DRI subscale, which only slightly matched the dimensions [89]. This raises the question of whether the extracted dimensions as well as the generic theoretical concepts identified by Galvin et al. and Titler and Pettit are applicable to psychiatric patients [7,10]. The only dimensions extracted that are not included in the RHDS, for example, are the social and organisational determinants. The relationship between individual factors and discharge readiness has been investigated in studies involving new mothers [31] and post-stroke individuals [93]. In a study on predictors of readiness for hospital discharge after birth by Malagon-Maldonado et al., social determinants such as three or more children, delivery mode, bottle-feeding, and the delivery of education were significant predictors of discharge readiness [64]. Among psychiatric patients, social determinants collected at admission, such as personal, demographic, and intellectual data, were significantly associated with post-treatment DRI scores [89]. One study showed that the type of diagnosis was associated with higher or lower readiness scores [99]. Thus, social and organisational determinants seem relevant for the assessment of discharge readiness, at least at the individual level for specific patient populations. It is questionable whether organisational and individual determinants are necessary and sufficient attributes or conditions to fully describe the concept of readiness to discharge, or whether they are relevant influencing factors to be assessed. Based on our data, there is a research gap that may be of great importance for the conceptualisation of readiness for discharge.

A generic concept and operational definition of discharge readiness across patient groups were provided for the first time in Galvin et al.’s conceptual analysis [10]. Most of the identified theoretical studies addressed a specific patient population. Although considered an advantage, as factors for concretising the dimensions could be derived for specific patient populations, the lack of transferability of the results can be seen as a disadvantage.

In some of the identified studies, emergency department visits, hospital readmission, and death within 30 days after discharge were reported as post-hospital outcomes when discharge readiness was assessed [30,35,41,71]. For instance, the registered nurse RHDS long- and short-form is an appropriate instrument to identify medical-surgical patients at risk of potential unplanned return to the hospital within 30 days [67]. A study of the predictors and effects of unplanned readmissions, emergency department visits, and death showed that being unready for discharge increased the risk of 30 day unplanned readmission and 30 day death [71]. These reported outcomes are consistent with the consequences of readiness for hospital discharge identified by Galvin et al., who additionally listed retaining control, autonomy, and dignity as well as feelings of safety, security, support, reducing the cost of care, satisfaction, and increased quality of life as consequences.

Another objective of this review was to identify the existing instruments for measuring discharge readiness. The RHDS is an instrument that has been developed for the purpose of assessing readiness for discharge and is psychometrically validated. Compared to the B-PREPARED, which measures readiness retrospectively at the time of discharge, readiness was captured prospectively using the RHDS and DRI [11,92,88]. Weiss et al. stated that the revised 21-item scale had acceptable items, scales, and reliability characteristics and encompassed a broad representation of people in the hospital. The scale and reliability properties were similar across postpartum mothers, adult medical-surgical patients, and parents of hospitalised children. Cronbach's alpha coefficient for the internal consistency of the 21-item instrument was 0.89 [11]. In translations of the forms of the RHDS, Cronbach's alpha for the Chinese translations was higher at 0.91 [76] and 0.97 [14] whereas translations in Turkish or French had lower internal validity than the original 21-item scale, with a Cronbach's alpha of 0.74 [12] and 0.8 [72], respectively. These translations and cultural adaptions of the RHDS scales for different populations highlight the relevance of adapting measurement tools with respect to the global population and differences in health systems. In an implementation study by Weiss et al., the effect of implementing a discharge readiness assessment in adult medical-surgical units on the 30-day return to hospital was investigated in a randomised clinical trial (READI). One general result of the implementation study was that the READI interventions (READI1: RN-RHDS/SF; READI2: RN-RHDS/SF and PT-RHDS/SF; READI3: RN-RHDS/SF and PT-RHDS/SF with cut-off scores) were not effective in reducing hospital readmission. In contrast, the combination of patient self-assessment and nurse assessment of discharge readiness (READI2) had been found to reduce the return to hospital in wards with high readmission rates [58]. The implementation process was evaluated by Costa et al. [106]. As part of the implementation study, nurses were interviewed in focus groups and their involvement in the discharge readiness assessment resulted in improved and earlier awareness of patients' discharge needs and greater patient/family involvement [107]. This result indicates that discharge readiness assessed from patients and nurses' points of view can be useful in avoiding negative patient outcomes and reinforcing the RHDS as a useful instrument for this purpose.

In contrast to the RHDS, there is a discrepancy in the reported purpose of the CTM in identified empirical studies, which did not involve its validation or development. On the one hand, the studies refer that CTM was developed by Coleman et al. to measure the quality of transition from hospital to home [100]. On the other hand, the CTM has been used in identified empirical studies to measure patient readiness for discharge [98,99]. Although the German expert standard discharge management indicates that discharge readiness is an outcome measure of discharge planning, the question arises as to whether the CTM, which was developed as a quality measurement instrument for transition, is inversely suitable for use in the assessment of discharge readiness [16]. The PREPARED questionnaire was originally designed to obtain feedback from community consumers on discharge planning activities [95] while the DPQ was designed to assess patients' and family members' perceptions of their needs for follow-up care [105]. In contrast, Sasanuma et al. demonstrated that the motor FIM and cognitive FIM subscales of the Functional Independence Measure (FIM) score, originally developed to assess a patient's functional independence, are predictors of readiness for discharge home by establishing a cut-off value [102].

### 4.1 Strengths and limitations

This study provides an overview of existing instruments measuring readiness for hospital discharge in hospitalised patients and systematically searched for and clustered theoretical concepts of discharge readiness, taking into account rigorous methodological standards for scoping reviews. Cross-cultural adoption and differences in translation and cultural adaptation, especially in relation to RHDS, have not been addressed in detail in our review as this was not a primary objective of our work.

One limitation of our study was that the included studies were not critically appraised. In addition, the applicability and acceptability of the theoretical concepts and assessment instruments in the context of acute care were not investigated in our research. Further limitations of our study include the focus on adults, limitations on the inclusion of studies in languages other than English or German, and the possible overlap of validation and empirical studies.

## 5 Conclusions

Patient readiness for hospital discharge is generally considered an indicator of discharge planning and a predictor of quality of discharge and transition of care. Systematic measurement of readiness for hospital discharge, particularly from the patient's perspective combined with the nurse's perspective, could be helpful in reducing negative outcomes such as hospital readmissions. This review identified studies and theoretical concepts that report associations between readiness for discharge and individual and provider-specific, often indication- or subgroup-specific, determinants and outcomes. However, there is a lack of high-quality studies on the social and organizational determinants of discharge readiness. A variety of instruments have been used to assess discharge readiness. However, due to different epistemological interests, the studies and instruments are also based on different definitions and theoretical constructs. This underscores the urgent need for a research program on readiness to discharge from hospital that not only considers readiness to discharge as an operational tool and indicator of hospital discharge planning, but also embeds readiness to discharge in a theoretical concept of continuity of care that also considers prehospital determinants as well as posthospital aspects such as outpatient structures and community resources within the context of a multisectoral care journey, with the aim of improving the safety and quality of discharge and transition.

## Funding

This research was funded by the 10.13039/501100003542Baden-Württemberg Ministry of Science, Research and the Arts as part of the DHBW FFL 2020 state research funding programme.

## Data availability statement

No data was used for the research described in the article.

## CRediT authorship contribution statement

**Hanna Feldbusch:** Conceptualization, Data curation, Formal analysis, Investigation, Methodology, Validation, Visualization, Writing – original draft. **Marita Schmidt:** Formal analysis, Writing – review & editing. **Eva Maria Steeb:** Conceptualization, Data curation, Formal analysis, Project administration, Resources. **Natalie Paschek:** Formal analysis. **Maren Nemesch:** Formal analysis. **Yannick Sartory:** Formal analysis. **Rebekka Brenner:** Formal analysis. **Stefan Nöst:** Conceptualization, Data curation, Formal analysis, Funding acquisition, Methodology, Project administration, Resources, Supervision, Visualization, Writing – review & editing.

## Declaration of competing interest

The authors declare that they have no known competing financial interests or personal relationships that could have appeared to influence the work reported in this paper.
