# Supplementary material for: Theoretical concepts and instruments for measuring hospital discharge readiness: A scoping review
Source: Heliyon. 2024 Feb 21;10(5):e26554. doi: 10.1016/j.heliyon.2024.e26554 (PMC10909674; doi:10.1016/j.heliyon.2024.e26554)
Supplement: Multimedia component 1 [file mmc1.docx]

**Additional file 1. Supplementary data**

**Table S1**

A summary of theoretical papers and reviews (e3) included in the review.

| Authors, Year | Study design | Geographic context | Target group | Definition/ operationalization/ dimensions/ influencing factors etc. of discharge readiness |
| --- | --- | --- | --- | --- |
| Blakey et al., 2017 (1) | Systematic review | UK | People being readmitted to hospital aged 65 and over | Experience of elderly persons with readmission to a hospital, among others, on the perception of readiness for discharge: Patients reported that they were discharged too soon or were not prepared to go home.  The feeling of not being ready to go home could be associated with (the fear of) readmissions. |
| Causey-Upton et al., 2019 (2) | Discussion paper | USA | Patients after total knee replacement | The definition for discharge readiness after total knee replacement is often related to pain control, knee function, walking distance, increasing independence with ambulation and ability to climb stairs in the literature.  Influencing Factors of discharge readiness after total knee replacement are:   - Education - Prerehabilitation and Rehabilitation - Social Support - Rehabilitation Setting (inpatient/ outpatient etc.) - Analgesia - Patient Characteristics (e.g., age, presurgical functional level, complications) |
| De Morton et al., 2008 (3) | Systematic review | Australia | Older acute medical patients | Independent mobility is a key factor in determining discharge readiness for older patients. |
| Fiore et al., 2012 (4) | 3-round Delphi process | International | Patients following colorectal surgery | Criteria to determine readiness for hospital discharge following colorectal surgery:   - Tolerance of oral intake - Recovery of lower gastrointestinal function - Adequate pain control with oral analgesia - Ability to mobilize and self-care - No evidence of complications or untreated medical problems   Specific endpoints are defined for each criterion. After these criteria are achieved, discharge may take place as soon as the patient has adequate postdischarge support and is willing to leave the hospital. If a stoma was constructed, the patient or family should have received training on stoma care or has outpatient training arranged. |
| Fiore et al., 2012 (5) | Systematic review | Australia | Patients following colorectal surgery | In the 156 studies reviewed, 70 different sets of criteria to indicate readiness for discharge were described. Most studies applied a combination of three or four criteria. The most frequently cited criteria were:   - Tolerability of oral intake (80%) - Return of bowel function (70%) - Adequate pain control (44%) - Adequate mobility (35%)   The endpoints for these criteria were generally poorly defined. |
| Galvin et al., 2017 (6) | Literature review | Ireland | Generic (no specific patient group) | Attributes, antecedents, and consequences of RHD were identified, providing the basis for an operational definition and conceptual framework of RHD.  Operational definition: RHD is both a state and a process. It is characterized by   - Physical stability, including functional ability, and competence to manage self-care at home - Adequate support to cope with multiple demands after leaving the hospital - Psychological ability, where the patient has become confident enough to manage the transition or process - Adequate information and knowledge to respond to common problems in the posthospital period. |
| Malagon-Maldonado, 2015 (7) | Dissertation  Manuscript 1: Systematic review  Manuscript 2: Discussion paper  Manuscript 3: Sample study | USA | Antepartum, intrapartum, and postpartum mothers | Development of a conceptual framework that encompasses readiness factors at all stages of pregnancy and the birth process and emphasizes the role of the nurse.  Five main themes in relation to factors associated with postpartum readiness in the antepartum, intrapartum, and postpartum periods:   - Maternal sociodemographic characteristics (e.g., age, chronic disease) - Prenatal characteristics (e.g., prenatal class and care) - Hospital/ perinatal factors (e.g., way of delivery, birth weight) - Provider characteristics (e.g., practitioner’s age, certifications) - Postpartum characteristics (e.g., feeding method, length of stay)   Significant predictors of RHD:   - Infant length of stay - Delivery of education - Difference between educational content received and content needed |
| Supattra Changsuphan et al., 2018 (8) | Systematic review | Thailand | Patients after total hip replacement (THR) | Five main themes influential to the readiness of THR patients for discharge:   - Physiological experiences (recovering mobility, physical safety) - Psychological experiences - Coping ability - Needs from the healthcare team (personal assessment, information need, multidisciplinary team) - Family support (after discharge) |
| Titler and Pettit, 1995 (9) | Literature review | USA | Generic (no specific patient group) | Readiness for discharge is part of discharge planning.  Definitions of discharge readiness:   - Readiness for discharge as a multifaceted, multistage concept that provides an estimate of patients’ and their family members’ ability to leave an acute care facility. - Discharge readiness as patients’ and families’ perception of being prepared or unprepared for hospital discharge.   Discharge readiness assessment implies evaluation of strengths and needs in five areas:   - Physiologic stability of the patient - Competency (cognitive and psychomotor) of the patient and family to carry out self-care management regimens - Perceived self-efficacy to carry out self-care management regimens - Availability of social support - Access to the health care system and community resources |

**Table S2**

A summary of development or validation studies (e1) included in the review.

| Author, Year | Study design | Geographic context | Study population | Data source | Aim | Assessment tool | Assessment perspective |
| --- | --- | --- | --- | --- | --- | --- | --- |
| Weiss et al., 2010 (10) | Validation study | USA | Medical-surgical patients (n=162); nurses (n=162) | Secondary data | Investigate the association between nurses' and patients' assessment of RHD in Terms of readmission and emergency department visits 30 days after discharge (post-discharge outcomes) | RHDS (21 items) | Self-reported; nurse-reported |
| Weiss and Piacentine, 2006 (11) | Validation study | USA | Medical-surgical patients (n=121); postpartum mothers (n=122); parents of hospitalized children (n=113) | Secondary data | Evaluation of the psychometric properties (validity and reliability) of the RHDS with 23 items | RHDS (23 items) --> revised RHDS (21 items) | Self-reported |
| Bobay et al., 2010 (12) | Validation study | USA | Medical-surgical patients (n=1892) | Primary data | To explore differences in perceptions of the quality of discharge teaching (QDT) and RHD and their relationship to postdischarge utilization of emergency department visits and readmissions across older age spectrum. Applicability of QDT und RHD assessment tools for older patients. | RHDS (21 items); dichotomous single item for RHD | Self-reported |
| Bobay et al., 2018 (13) | Validation study | USA | Matched nurse-medical-surgical patients’ pairs (sample 1 n= 162 pairs sample 2 n=154 pairs) | Primary data | Determine the psychometric properties of the long and short forms of the RN-RHDS incl. Reliability, factor structure and predictive validity | RN-RHDS (21 items); RN-RHDS/SF (8items) | Nurse-reported |
| Bull, 1994 (14) | Validation study | USA | Health care professionals (n=38); Elders (n=25; n=42) | Primary data | Evaluation of the psychometric properties of DRT and description of the scale development of the DPQ | DPQ | Self-reported |
| Buszko et al., 2017 (15) | Validation study | Poland | Patients with coronary artery disease (n=201) | Primary data | To validate a new self-reported questionnaire assessing the readiness of patients for hospital discharge | RHD-MIS | Self-reported |
| Chen et al., 2019 (16) | Validation study | China | People living with HIV (n=213) | Primary data | Validation of the reliability and factor structure of the Chinese version of the RHDS (RHDS-CH) for people living with HIV. | RHDS-CH (23 items) | Self-reported |
| Coleman et al., 2005 (17) | Validation study | USA | Adult patients (n=200) | Primary data | To develop and test a self-reported measure of the quality-of-care transitions (CTM) (…). | CTM (15 items) | Self-reported |
| Fenwick, 1979 (18) | Not evaluable | UK | Rehabilitation patients (n=not evaluable) | Primary data | Development and application of an interdisciplinary tool for assessing patients' readiness for discharge in the rehabilitation setting | Interdisciplinary tool for assessing patients' readiness for discharge in the rehabilitation setting | Third party-reported |
| Gooding et al., 2021 (19) | Observational study | South Africa | Mothers practicing continuous kangaroo mother care(n=200) | Primary data | Development and application of a tool to measure RHD of mothers practicing continuous kangaroo mother care | Maternal Discharge Readiness Tool (22 items) | Self-reported |
| Graumlich et al., 2008 (20) | Validation study | USA | Home discharged patients | Primary data | Evaluation of the psychometric properties of the B-PREPARED instrument to measure preparedness for hospital discharge from patient's perspective | Brief PREPARED instrument | Self-reported |
| Grimmer and Moos, 2001 (21) | Validation study | Australia | Different samples for development, piloting and validation trial from nurses (n=8); hospital staff (n=26); patients (n=26; n=50; n=834); | Primary data | To describe the development, validity, and application of PREPARED for obtaining feedback from community consumers of discharge planning activities | PREPARED | Self-reported |
| Hariati et al., 2020 (22) | Validation study | Indonesia | Mothers of low-birth-weight infants (n=146) | Primary data | Cross-cultural adaption and psychometric validation of the original version of the RHDS-Parent into Bahasa Indonesia. | Bahasa-RHDS-Parent (22 items) | Nurse-reported |
| Hariati et al., 2020 (23) | Observational study | Indonesia | Mothers of low-birth-weight infants (n=139) | Primary data | Description of Indonesian mothers' discharge readiness, including translation of the RHDS into an Indonesian version. | Indonesian version of the RHDS (29 items) | Self-reported; nurse-reported |
| Huiling Zao et al., 2016 (24) | Validation study | China | Laryngectomy patients (n=202) | Primary data | Translation of the RHDS into Chinese and assessment of the psychometric properties of this scale on laryngectomy patients in China | Chinese version of the RHDS (22 items) | Self-reported |
| Kaya et al., 2018 (25) | Validation study | Turkey | Internal medicine patients (n=1579) | Primary data | Evaluation of the psychometric properties (validity and reliability) of the Turkish version of the RHDS/SF | Turkish version of the RHDS/SF (8 items) | Self-reported |
| Kleinknecht-Dolf et al., 2019 (26) | Validation study | Switzerland | Home discharged patients | Primary data | Examination of patients' RHD in Switzerland and evaluation of the practicable applicability of the German speaking RHDS/SF (incl. translation of the RHDS/SF into German) | German speaking RHDS/SF (8 items) | Nurse-reported |
| Mabire et al., 2015 (27) | Validation study | USA | Older medical-surgical patients (n=998) | Secondary data | Evaluation of the psychometric properties of RHDS for older people and development of a RHDS/SF for use with older adults | RHDS (23 items) and RHDS-OP-SF (9 items) | Self-reported |
| Mabire et al., 2015 (28) | Validation study | Switzerland | Older medical patients (n=265) | Primary data | Evaluation of the psychometric properties (reliability and factor structure) of the RHDS-Fr (incl. translation of the RHDS into French) | RHDS-Fr (20 items) | Self-reported |
| Nagorska and Darmochwal-Kolarz, 2019 (29) | Validation study | Poland | Postpartum women (n=168) | Primary data | Evaluation of the psychometric properties of the Polish version of the RHDS for subjective assessment of the bio-psycho-physical status of women after childbirth. | Polish version of the RHDS for postpartum mothers (23 items) | Self-reported |
| Potkin et al., 2005 (30) | Validation study | International | Schizophrenic or schizoaffective patients (n=500) | Secondary data | To describe the psychometric properties of the RDQ. | RDQ (6 items) | Third party-reported |
| Weiss et al., 2014 (31) | Validation study | USA | Medical-surgical patients (n=254); nurses (n=54) | Primary data | To validate patient and nurse short forms for discharge readiness assessment and their associations with 30-day readmission and emergency department visits. | PT-RHDS/SF (8 items); RN-RHDS/SF (8 items) | Self-reported; nurse-reported |
| Weiss et al., 2006 (32) | Validation study | USA | Postpartum women (n=1 462) | Secondary data | To assess the psychometric properties of the PRDBS. | PRDBS (9 items); Single item | Self-reported |
| Hodgins et al., 2020 (33) | Validation study | Canada | Medical-surgical patients (n=42) | Primary data | To pilot the proposed protocol, to assess psychometric properties of instruments used to measure perceived readiness for discharge and postdischarge coping, (…). | Modified RHDS/SF (8 items) for use in predischarge and postdischarge interviews | Self-reported |
| Mixon et al., 2016 (34) | Observational study | USA | Adults hospitalized for cardiovascular diagnoses (n=1 239) | Primary data | To determine whether the B-PREPARED and CTM-3 measures were predictive of readmission or death, as compared to the LACE index, and to determine the additional predictive and discriminative ability gained from administering the B-PREPARED and CTM-3 measures (...). | B-PREPARED (11-item) and CTM-3 (3 item) | Self-reported |
| Natthawan Suwan et al., 2018 (35) | Observational study | Thailand | Older patients hospitalized with an exacerbation pf COPD (n=207) | Primary data | To determine the predictive power of factors on readiness for hospital discharge among older persons with COPD. | Thai RHDS-Adult Form (23 items) | Self-reported |
| Sasanuma et al.,2015 (36) | Observational study | Japan | Hospitalized patients undergoing cardiac rehabilitation (n=949) | Secondary data | To verify whether the Functional Independence Measure score, and its subscale motor (FIM^TM^) and cognitive (FIM^TM^), during inpatient cardiac rehabilitation can be a predictor of a patient’s readiness for home discharge by establishing an FIM^TM^ cutoff value. | FIM^TM^ instrument (18 items) | Third party-reported |
| Weiss et al., 2019 (37) | Experimental study | USA; Saudi Arabia | Medical-surgical patients (n= 144 868) | Primary data | To determine the effect of unit-based implementation of readiness evaluation and discharge intervention protocols on readmissions and emergency department or observation visits. | RHDS/SF (8 items) | Varies depending on the protocol used (self-reported; nurse-reported) |
| Wong et al., 1999(38) | Validation study | Canada | Patients following total hip replacement (n=50) | Primary data | To investigate the home-readiness and recovery pattern of patients after total hip replacement surgery. In particular, to test the reliability and validity of the PTHRDSS (…). | Modified PTHRDSS (9 items) | Third party-reported |
| Hogarty and Ulrich, 1972 (39) | Validation study | USA | Chronically hospitalized and recently admitted schizophrenic in-patients (n=2 000) | Primary data | Evaluation of the psychometric properties of DRI and Description of the scale development. | DRI | Third party-reported |

**Table S3**

Summary of included empirical studies with discharge readiness as primary outcome or key concept (e2).

| Author, Year | Study design | Geographic context | Study population | Aim | Measurement tool | Data source | Purpose of the measurement | Assessment perspective |
| --- | --- | --- | --- | --- | --- | --- | --- | --- |
| Qian et al., 2021 (40) | Observational study | China | Patients discharged with tubes (n=161) | To investigate the RHD of patients discharged with tubes from the department of hepatobiliary surgery and to explore influencing factors. | Modified Chinese version of the RHDS (23 items) | Primary data | Assess patients' RHD | Self-reported |
| Hu et al., 2020 (41) | RCT | China | Patients admitted undergoing kidney transplantation (n=220) | Evaluation of the effectiveness of an innovative transitional care program in improving discharge readiness (…). | Chinese version of the RHDS (22 items) | Primary data | Assess patients' RHD | Self-reported |
| Andrew et al., 2018 (42) | Observational study | Australia | Patients with a stroke or TIA being discharged to community (n=200) | To describe the quality of discharge planning received by patients discharged home from acute care, identify factors associated with a positive discharge experience, and assess the influence of discharge quality on outcomes. | PREPARED questionnaire | Primary data | Assess quality of discharge care planning | Self-reported |
| Archer et al., 1980 (43) | Observational study | USA | Patients of a short-term residential psychiatric treatment program (n=88) | To examine the relationship between staff related discharge readiness and patient personality, demographic, and intellectual variables. | DRI | Primary data | Assess patients' discharge readiness | Third party-reported |
| Aviram et al., 1995 (44) | Observational study | USA | Hospitalized psychiatric patients (n=2 775) | Using the state of New Jersey as an example, patients ready for discharge will be described, their service needs discussed and the difficulties in trying to access alternative community care services highlighted. | DPP status or the LOC designations | Secondary data | Identify discharge-ready patients |  |
| Bahr et al., 2020 (45) | Observational study | USA | Medical-surgical patients (n=18 203) | To examine the association of continuity in nurse assignment with return to hospital, including exploration of the mediating pathway through patient readiness for discharge and moderating effects of unit environment and unit nurse characteristics. | PT-RHDS/SF (8 items) and RN-RHDS/SF (8 items) | Secondary data | Assess patients' RHD | Self-reported; nurse-reported |
| Baksi et al., 2020 (46) | Observational study | Turkey | Post craniotomy individuals (n=150) | To investigate the relationship between demographic and clinical characteristics of post craniotomy individuals and the factors likely to influence their discharge readiness | Turkish version of the RHDS/SF (8 items) | Primary data | Assess patients' RHD | Self-reported |
| Blumer et al., 2021 (47) | Quasi-experimental study | Canada | Patients hospitalized for heart failure (n=986) | To assess the effect of transitional care on patient-reported outcomes (e.g., discharge preparedness) in patients hospitalized for heart failure. | B-Prepared telephone survey instrument (11 items) | Primary data | Assess patients' discharge preparedness | Self-reported |
| Bobay et al., 2021 (48) | Qualitative study | USA; Saudi Arabia | Nurses using the implemented READI study protocols to assess readiness for discharge (n=135) | To describe clinical nurses' experiences with practice change associated with participation in a multisite nursing translational research study (READI) implementing new protocols for hospital discharge readiness assessment | Three different protocols | Primary data | Assess patients' RHD | Varies depending on the protocol used (self-reported; nurse-reported) |
| Braet et al., 2016 (49) | Observational study | Belgian | Patients with heart failure, pneumonia, or total hip/ knee arthroplasty (n=233) | To evaluate variations in the quality of transitions across groups of patients and across hospitals with high and low readmission rates and to study the impact of transitions on postdischarge outcomes. | Dutch translation of the CTM | Primary data | Assess patients' readiness for discharge | Self-reported |
| Brent and Coffey, 2013 (50) | Observational study | Ireland | Patients following hip fracture surgery (n=50) | To examine patient's perception of their readiness for discharge post hip fracture and to establish whether relationships existed between patient's perception of their readiness for discharge and demographic variables. | RHDS (23 items) | Primary data | Assess patients' RHD | Self-reported |
| Caminsky et al., 2021 (51) | Observational study | Canada | Patients undergoing elective colorectal surgery (n=73) | To compare time to readiness for discharge by set criteria and actual length of stay in a recovery pathway and to identify reasons for delayed hospital discharge. | Set Criteria (Fiore et al. 2012) | Primary data | Identification of day of discharge readiness | Third party-reported |
| Celio et al., 2019 (52) | Observational study | Switzerland | Patients following colorectal resection (n=138) | To analyze reasons and risk factors of discharge delay (effective day of discharge > postoperative day of readiness for discharge) | Set Criteria (Fiore et al. 2012) | Primary data | Identification of day of discharge readiness | Third party-reported |
| Clark et al., 1997 (53) | Observational study | Australia | Older Patients aged 60 years and over (n=76) | To examine the interface between acute hospital care and return to home in relation to elderly patients' perceived ability and preparedness to cope at home. | Two developed questionnaires | Primary data | Assess the perceived level of preparedness for coping at home (among other purposes) | Self-reported |
| Coffey and McCarthy, 2013 (54) | Observational study | Ireland | Older patients over 65 years (n=335) | To examine older patients’ perception of their readiness for discharge from hospital to home and use of community supports postdischarge. | RHDS (23 items) | Primary data | Assess patients' RHD | Self-reported |
| Congdon, 1994 (55) | Qualitative study | USA | Older patients (n=8) | To describe the elderly person's hospital discharge experience |  | Primary data |  |  |
| Dols et al., 2020 (56) | Quasi-experimental study | USA | Single-organ liver transplant recipients (n=86) | To compare the 30-day readmission of transplant recipients for 1-year prior and 1-year following the implementation of nurse-led education | 10-point Activities poster (Readiness for discharge patient goals and activities) | Primary data | Assess and monitor patients' readiness for discharge | Self-reported; nurse-reviewed |
| Du Plessis et al., 2018 (57) | Observational study | South Africa | Patients with spinal cord injury (n=50) | To compare the perception of patients with spinal cord injury and physiotherapists regarding RHD. | RHDS (21 items) | Primary data | Assess patients' RHD | Self-reported; physiotherapist-reported |
| Rotvig et al., 2021 (58) | Observational study | Denmark | Patients with cardiac diseases (n=13 114) | To investigate whether RHD predicts readmission and mortality within 1-year post-discharge, as well as the association between physical stability, adequate support, psychological ability, and adequate information and knowledge and RHD. | One validated single item; Measurement of the dimensions of RHD via Physical Component Summary of the Short Form-12, Edmonton Symptom Assessment Scale, patient-reported questions from the DenHeart survey | Primary data | Assess patients' RHD and dimensions of RHD | Self-reported |
| Wallace et al., 2018 (59) | Mixed methods | USA | Medical-surgical patients (n=70) | To evaluate an interactive tool designed to help patients communicate their social resources supportive of home recovery to health care providers. | RHDS (22 items) | Primary data | Assess patients' perceived readiness for discharge | Self-reported |
| Grimmer et al., 2006 (60) | Quasi-experimental study | Australia | Older patients aged at least 60 years (n=317) | Effectiveness of a checklist that assists patients to transition safely and sustainably from hospital to home. | PREPARED questionnaire; some qualitative questions on aspects of RHD | Primary data | Assess the quality of patients' discharge preparation from hospital | Self-reported |
| Handy, 1974 (61) | Observational study | USA | Hospitalized schizophrenic men (n=465) | To determine whether judgements of release readiness are related to discharge. | Constructed questionnaire (by the author) | Primary data | Judgment on a patient's readiness or nonreadiness for release | Third party-reported |
| Harrison et al., 2020 (62) | Observational study | USA | Adult patients (n=179) | To evaluate how assessing patient readiness for discharge effects discharge outcomes. | Adapted checklist (12 items) based on RHDS (23 items) | Primary data | Assess patients' RHD | Self-reported |
| Harrison et al., 2016 (63) | Observational study | USA | Adult patients (n=163) | To determine how often patient-reported barriers to discharge on admission were resolved by discharge and to explore associations between barriers and readmission. | Adapted checklist (12 items) based on RHDS (23 items) | Primary data | Assess patients' RHD | Self-reported |
| Heine et al., 2004 (64) | Qualitative study | Australia | Patients following total hip replacement (n=5) | To gain an understanding of the experiences of people undergoing a total hip replacement, in relation to their pending discharge. |  | Primary data |  |  |
| Hogarty, 1968 (65) | Observational study | USA | Chronic schizophrenic patients (n=206) | To examine hospital differences in the release of discharge ready chronic schizophrenic patients into outpatient care. | DRI | Primary data | Assess patients' discharge readiness | Third party-reported |
| Hydzik et al., 2021 (66) | Observational study | Poland | Participants diagnosed with myocardial infarction (n=102) | To evaluate the associations between patients' RHD after myocardial infarction, acceptance of illness, social, demographic, and clinical factors | RHD-MIS (23 items) | Primary data | Assess the readiness for discharge of patients after myocardial infarction | Self-reported |
| Jack et al., 2009 (67) | RCT | USA | Hospitalised adults (n=749) | To test the effects of an intervention designed to minimize hospital utilization after discharge. | Single item: " How prepared were you to leave the hospital?" | Primary data | Assess patients' readiness for discharge | Self-reported |
| Yang et al., 2020 (68) | Observational study | China | Colorectal cancer patients (n=130) | To ascertain the status quo of perceived RHD in colorectal cancer patients and identify the variables that affect patients' perceptions about their readiness for discharge (n=130) | Chinese version of the RHDS (22 items) | Primary data | Assess patients' RHD | Self-reported |
| Kaya et al., 2018 (69) | Observational study | Turkey | Internal-medicine patients (n=1 601) | To determine the variables that affect patients' perceptions about their readiness for discharge and to elucidate the effects of these perceptions on patient outcomes | Turkish version of the RHDS/SF (8 items) | Primary data | Assess patients' RHD | Self-reported |
| Kelly et al., 1998 (70) | Observational study | Canada | Hospitalized psychiatric patients (n=327) | To determine if there are any potential opportunities for patients to be discharged earlier and to determine what factors are responsible for delays in discharge. | DRI | Primary data | Assess patients' discharge readiness or their need for continuing stay in the psychiatric setting | Third party-reported |
| Kleinpell, 2004 (71) | RCT | USA | Older patients (≥65 years) being hospitalized in the ICU (n=100) | To pilot test an ICU-based nursing screening intervention to assist in determining the discharge needs and outcomes of critically ill elderly patients. | DPQ (51 items) | Primary data | Assess discharge needs | not reported |
| Kosobucka et al., 2020 (72) | Observational study | Poland | Myocardial infarction patients treated with percutaneous coronary intervention (n=213) | To analyze the relationship between RHD and adherence to treatment at follow-up in myocardial infarction patients. | RHD-MIS (23 items) | Primary data | Assess the readiness for discharge of patients after myocardial infarction | Self-reported |
| LaManna et al., 2016 (73) | Mixed methods | USA | Older adults (n=96) | To determine whether personal and community transition conditions impacted the early and intermediate post-discharge outcomes in a sample of older adults with diabetes. | RHDS (23 items) | Primary data | Assess patients' RHD | Self-reported |
| Lau et al., 2016 (74) | Observational study | Canada | Internal-medicine patients (n=495) | To examine predictors of readmission or death among patients discharged. | Single item | Secondary data | Assess patients' discharge readiness | Self-reported |
| Liang et al., 2021 (75) | Observational study | China | Patients with coronary heart disease who had received a coronary stent placement(n=153) | To explore the association between RHD and 30-day adherence to treatment | Chinese version of the RHDS (22 items) | Primary data | Assess patients' RHD | Self-reported |
| Mabire et al., 2015 (76) | Observational study | Switzerland | Older medical patients aged 65 years and over (n=196) | To describe the usual discharge planning process and to investigate the relationship between the comprehensiveness of the nursing discharge planning process and the patients RHD and unplanned health care utilization after discharge. | RHDS (22 items) | Primary data | Assess patients' RHD | Self-reported |
| Mabire et al., 2019 (77) | Observational study | Switzerland | Nurses (n=1 833) and hospitalized patients (n=1 755) | To explore the associations between structure-individual characteristics and process-related factors and patient RHD. | Single item | Secondary data | Assess patients' RHD | Self-reported |
| Malagon-Maldonado et al., 2017 (78) | Observational study | USA | Postpartum women (n=185) | To identify antepartum, intrapartum, and postpartum factors, including nursing educational practices, most predictive of postpartum mothers' perception of RHD. | RHDS-New Mother Form (22 items) | Primary data | Assess new mothers' perceptions of RHD | Self-reported |
| Manges et al., 2020 (79) | Observational study | USA | Discharge events consisting of the patient and their team (n=64) | To determine the convergence of interprofessional team shared mental models of hospital discharge readiness and to identify factors associated with these assessments. | RHDS/SF (8 items) | Primary data | Assess patients' RHD | Self-reported; team-reported |
| Meng et al., 2020 (80) | Observational study | China | Patients diagnosed with anxiety disorders (n=373) | To explore the factors of RHD and the effect of patient reported RHD on postdischarge outcomes. | RHDS (23 items) | Primary data | Assess patients' RHD | Self-reported |
| Meo et al., 2020 (81) | Qualitative study | USA | Patients experiencing prolonged hospitalization while being "medically ready" or stable for discharge (n=not reported) | To describe both the challenges and the opportunities in the care of these patients and to offer recommendations for a path forward. |  | Secondary data |  |  |
| Mess et al., 2021 (82) | Observational study | Poland | Hospitalized women with cancer (n=100) | To assess the emotional condition of women with cancer | C-HOBIC (8 items) | Primary data | Assess women’s readiness for discharge | Self-reported |
| Middleton et al., 2004 (83) | Observational study | Australia | Patients following carotid endarterectomy (n=133) | To determine patients' knowledge before admission about how many days they were likely to be hospitalized and, after discharge, to determine patients' readiness to leave hospital. | Post-operative questionnaire | Primary data | Retrospectively assess patients' readiness to leave the hospital. | Self-reported |
| Nurhayati et al., 2019 (84) | Observational study | Indonesia | Abdominal surgery patients (n=96) | To examine the level of perception of the quality of discharge teaching and its associations with the RHD among surgical patients in acute care hospitals. | Indonesian version of the RHDS (20 items) | Primary data | Assess patients' RHD | Self-reported |
| Opper et al., 2019 (85) | Quasi-experimental study | USA | Health team members (n=105); patient (n=413) | To determine whether a redesigned health team communication process related to hospital discharge improves communications and collaboration between nurses and physicians; patient experiences od discharge care as measured by quality of discharge teaching, readiness for discharge, and postdischarge coping difficulty; and the rate of readmissions and emergency department visits within 30 days postdischarge. | PT-RHDS/SF (8 items), RN-RHDS/SF (8 items), MD-RHDS/SF (8 items) | Primary data | Assess patients' RHD | Self-reported; nurse-reported; physician-reported |
| Peyrovi et al., 2016 (86) | Quasi-experimental study | Iran | Mothers and their premature infants (n=80 pairs) | To examine the effect of empowerment program on "perceived readiness for discharge" of mothers of premature infants at the time of discharge. | Parent Discharge Readiness questionnaire (14 items) | Primary data | Assess mothers' perceived readiness for discharge | Self-reported |
| Presciutti et al., 2020 (87) | Observational study | USA | Survivors of cardiac arrest (n=163) | To evaluate associations between provider-patient communication, Readiness for discharge, and patients' illness perceptions with post-arrest QOL. | RHDS/SF (8 items) | Primary data | Assess patients' RHD | Self-reported |
| Rodrigue et al., 2017 (88) | Observational study | Canada | Hospitalized trauma patients 65 years and older (n=33) | To describe the experiences of hospitalized trauma patients 65 years and older who are discharged home | PREPARED questionnaire (50 items) | Primary data | Assess patients' discharge preparedness | Self-reported |
| Saunders et al., 2021 (89) | Mixed methods | Canada | Adult inpatients (n=25); their caregivers (n=14) who received a palliative care consultation and transitioned to home-based palliative care | To gain insight into how patients experience three domains, discharge readiness, transition quality, and discharge-coping, during hospital-to-home transitions. | RHDS/SF (8 items) | Primary data | Measure patient or caregiver readiness to be discharged from the hospital | Self-reported and/or caregiver-reported |
| Schaefer et al., 1990 (90) | Observational study | USA | Older surgical patients 65 years and older (n=25) | To study perceptions o readiness for discharge and the need for at-home, family, and community resources among persons aged 65 years and older. | Questionnaire developed by Anderson and Smith | Primary data | Assess patients' readiness and 5 factors of internal readiness | Self-reported |
| Schmocker et al., 2015 (91) | Observational study | USA | Medical-surgical patients (n=318) | To examine the association of readiness for discharge with patient satisfaction and readmission. | Survey question: "Did your feel ready for discharge?" | Secondary data | Assess patients' readiness for discharge | Self-reported |
| Schneider and Howard, 2017 (92) | Quasi-experimental study | USA | Patients with a diagnosis of stroke or transient ischemic attack (n=86) | To examine differences in discharge readiness and postdischarge coping in patients admitted for stroke after the use of individualized postdischarge information/ education provided via a technology package compared with current standard discharge teaching methods. | RHDS (no further information) | Primary data | Assess patients' discharge readiness | Self-reported |
| Siow et al., 2019 (93) | Observational study | China | Emergency medicine ward patients (n=184) | To examine the factors and outcomes associated with patients' RHD in an emergency medicine ward setting. | Chinese version of the RHDS (22 items) | Primary data | Assess patients' RHD | Self-reported |
| Sriprasong et al., 2009 (94) | Observational study | Thailand | Post-acute myocardial infarction patients (n=180) | To test, one-month post-hospitalization, the effects of discharge readiness (…) on functional status of individuals who had experiences acute myocardial infarction. | Thai translation of the RHDS-Adult Form (23 items) | Primary data | Assess patients' RHD | Self-reported |
| Verhaegh et al., 2019 (95) | Qualitative study | Netherlands | Chronically ill patients (n=23) | To explore chronically ill patients' experiences and perceptions of being discharged to home and then acutely readmitted to the hospital to identify the potential impact on future care transition interventions. |  | Primary data |  |  |
| Wallace et al., 2018 (96) | Observational study | USA | Veterans (n=70) | To better understand whether and how patient- and nurse-assessed readiness for discharge is related to patient experiences after discharge. | Pt-RHDS (23-items), RN-RHDS (23-items) | Secondary data | Assess patients' RHD | Self-reported; nurse-reported |
| Wallace et al., 2016 (97) | Observational study | USA | Medical-surgical veterans (n=70) | To better understand whether and how readiness for hospital discharge varies by personal characteristics, including health literacy. | Pt-RHDS (23-items), RN-RHDS (23-items) | Secondary data | Assess patients' RHD | Self-reported; nurse-reported |
| Wang et al., 2021 (98) | Observational study | China | Diabetic foot ulcer patients (n=179) | To investigate the correlation of RHD, the chronic illness resources, and postdischarge outcomes of diabetic foot ulcer patients, which can be help for discharged patient rehabilitation. | Chinese version of the RHDS (12 items) | Primary data | Assess patients' RHD | Self-reported |
| Wang et al., 2021 (99) | Observational study | China | Patients with depression (n=367) | To examine patient reported RHD and its factors among patients with depression. | (Modified) Chinese version of the RHDS (23 items) | Primary data | Assess patients' discharge readiness | Self-reported |
| Weiss et al., 2004 (100) | Observational study | USA | New mothers (n=1 192) | To compare the sociodemographic characteristics and readiness for discharge of new mothers and their newborns at 3 discharge time intervals, and to determine which variables were associated with postpartum length of stay. | RHDS-After Birth Scale (9 items) | Primary data | Assess mothers' perceived readiness for discharge | Self-reported |
| Weiss and Lokken, 2009 (101) | Observational study | USA | Postpartum mothers (n=141) | To identify predictors and outcomes of postpartum mothers' perceptions of their RHD. | RHDS-New Mother Form (22 items) | Primary data | Assess mothers' perceived readiness for discharge | Self-reported |
| Weiss et al., 2007 (102) | Observational study | USA | Medical-surgical patients (n=147) | To identify predictors and outcomes of adult medical-surgical patients' perceptions of their RHD. | RHDS-Adult Form (22-items) | Primary data | Assess patients' perceptions of RHD | Self-reported |
| Weiss et al., 2011 (103) | Observational study | USA | Medical-surgical patients (n=1 892) | To determine the impact of unit-level nurse staffing on quality of discharge teaching, patient perception of discharge readiness, and postdischarge readmission and emergency department visits, and cost-benefit of adjustments to unit nurse staffing. | RHDS (21 items) | Hybrid data use | Assess patients' perceptions of RHD | Self-reported |
| Xiong et al., 2021 (104) | Observational study | China | Patients with major depressive disorder (n=224) | To investigate the patient reported RHD and its influencing factors among Chinese adult patients with major depressive disorder | Chinese version of the RHDS (22 items) | Primary data | Assess patients' perceptions of RHD | Self-reported |
| Yanıkkerem et al., 2018 (105) | Observational study | Turkey | Postpartum women (n=610) | To evaluate the factors affecting readiness for discharge and perceived social support after childbirth. | Turkish version of the RHDS-New Mother Form (23 items) | Primary data | Assess postpartum women's readiness for discharge | Self-reported |
| Zhang et al., 2021 (106) | Observational study | China | Cataract patients (n=192) | To explore the interaction mechanism of cataract patients' quality of discharge teaching, discharge readiness and post-discharge outcomes using structural equation model analysis. | Chinese version of the RHDS (22 items) | Primary data | Assess patients' RHD | Self-reported |
| Zhao et al., 2020 (107) | Observational study | China | Laryngeal cancer patients (n=212) | To identify factors influencing RHD among Chinese patients having undergone a laryngectomy and to provide evidence for developing future processes. | Chinese version of the RHDS (23 items) | Primary data | Assess patients' perceptions of RHD | Self-reported |

*Note:* RHD = Readiness for hospital discharge

References

1. Blakey EP, Jackson D, Walthall H, Aveyard H. What is the experience of being readmitted to hospital for people 65 years and over? A review of the literature. CONTEMP NURSE 2017; 53(6):698–712.

2. Causey-Upton R, Howell DM, Kitzman PH, Custer MG, Dressler EV. Factors Influencing Discharge Readiness After Total Knee Replacement. ORTHOP NURS 2019; 38(1):6–16.

3. de Morton NA, Berlowitz DJ, Keating JL. A systematic review of mobility instruments and their measurement properties for older acute medical patients. HEALTH QUAL LIFE OUTCOMES 2008; 6:44.

4. Fiore JF, Browning L, Bialocerkowski A, Gruen RL, Faragher IG, Denehy L. Hospital discharge criteria following colorectal surgery: a systematic review. Colorectal disease 2012; 14(3):270–81.

5. Fiore JF, Bialocerkowski A, Browning L, Faragher IG, Denehy L. Criteria to determine readiness for hospital discharge following colorectal surgery: an international consensus using the Delphi technique. Diseases of the colon and rectum 2012; 55(4):416–23.

6. Galvin EC, Wills T, Coffey A. Readiness for hospital discharge: A concept analysis. Journal of Advanced Nursing 2017; 73(11):2547–57.

7. Malagon-Maldonado G. Antepartum, intrapartum, and postpartum predictors of readiness for hospital discharge and post-discharge outcomes. Dissertation 2015. University of San Diego. https://doi.org/10.22371/07.2015.011

8. Supattra Changsuphan, Puangpaka Kongvattananon, Chomchuen Somprasert. Patient readiness for discharge after total hip replacement: An integrative review. Journal of Health Research 2018; 32(2): 164-171.

9. Titler MG, Pettit DM. Discharge readiness assessment. J CARDIOVASC NURS 1995; 9(4):64–74.

10. Weiss M, Yakusheva O, Bobay K. Nurse and patient perceptions of discharge readiness in relation to postdischarge utilization. MED CARE 2010; 48(5):482-6.

11. Weiss ME, Piacentine LB. Psychometric properties of the Readiness for Hospital Discharge Scale. J NURS MEAS 2006; 14(3):163–80.

12. Bobay KL, Jerofke TA, Weiss ME, Yakusheva O. Age-related differences in perception of quality of discharge teaching and readiness for hospital discharge. GERIATR NURS 2010; 31(3):178–87.

13. Bobay KL, Weiss ME, Oswald D, Yakusheva O. Validation of the Registered Nurse Assessment of Readiness for Hospital Discharge Scale. NURS RES 2018; 67(4):305–13.

14. Bull MJ. A discharge planning questionnaire for clinical practice. APPL NURS RES 1994; 7(4):193–9.

15. Buszko K, Kosobucka A, Michalski P, Pietrzykowski Ł, Jurek A, Wawrzyniak M et al. The readiness for hospital discharge of patients after acute myocardial infarction: a new self-reported questionnaire. Medical Research Journal 2017; 2(1):20–8.

16. Chen C, Zhang X, Tang C, Xiao X, Tao Z, Wang H. Psychometric properties of the Chinese Version of the Readiness for Hospital Discharge Scale for people living with HIV. International journal of nursing sciences 2019; 7(2):220–7.

17. Coleman EA, Mahoney E, Parry C. Assessing the quality of preparation for posthospital care from the patient's perspective: the care transitions measure. MED CARE 2005; 43(3):246–55.

18. Fenwick AM. An interdisciplinary tool for assessing patients' readiness for discharge in the rehabilitation setting. Journal of Advanced Nursing 1979; 4(1):9–21

19. Gooding C, Lavin T, van Rooyen E, Bergh A-M, Preen DB. Evaluating Maternal Discharge Readiness in Kangaroo Mother Care. Indian pediatrics 2021.

20. Graumlich JF, Novotny NL, Aldag JC. Brief scale measuring patient preparedness for hospital discharge to home: Psychometric properties. J HOSP MED 2008; 3(6):446–54.

21. Grimmer K, Moss J. The development, validity and application of a new instrument to assess the quality of discharge planning activities from the community perspective. INT J QUAL HEALTH CARE 2001; 13(2):109–16.

22. Hariati S, McKenna L, Lusmilasari L, Reisenhofer S, Sutomo R, Febriani ADB et al. Translation, Adaptation and Psychometric Validation of the Indonesian Version of the Readiness for Hospital Discharge Scale for Parents of Low Birth Weight Infants. J PEDIATR NURS 2020; 54:e97-e104.

23. Hariati S, Sutomo R, Lusmilasari L, Febriani ADB, Kadar K. Discharge readiness of Indonesian mother with preterm infant in NICU. ENFERM CLIN 2020; 30:234–7.

24. Huiling Zhao, Xianqiong Feng, Rong Yu, Deying Gu, Xiaoqin Ji. Validation of the Chinese Version of the Readiness for Hospital Discharge Scale on Patients Who Have Undergone Laryngectomy. J NURS RES 2016; 24(4):321–8.

25. Kaya S, SAIN GUVEN G, Teleş M, Korku C, AYDAN S, Kar A et al. Validity and reliability of the Turkish version of the readiness for hospital discharge scale/short form. Journal of Nursing Management 2018; 26(3):295–301.

26. Kleinknecht-Dolf M, Lendner I, Müller R, Horlacher K, Martin JS, Spirig R. Einschätzung der Austrittsbereitschaft von Patienten in akutsomatischen Spitälern in der Schweiz durch Pflegefachpersonen: Eine Pilotstudie und Querschnittserhebung mit der deutschsprachigen Übersetzung der Kurzform der "Readiness for Hospital Discharge Scale". PFLEGEWISSENSCHAFT 2019; 21(1/2):30–40.

27. Mabire C, Coffey A, Weiss M. Readiness for Hospital Discharge Scale for older people: psychometric testing and short form development with a three country sample. Journal of Advanced Nursing 2015; 71(11):2686–96.

28. Mabire C, Lecerf T, Büla C, Morin D, Blanc G, Goulet C. Translation and psychometric evaluation of a French version of the Readiness for Hospital Discharge Scale. J CLIN NURS 2015; 24(19-20):2983–92.

29. Nagorska M, Darmochwal-Kolarz D. The adaptation of Polish version of the Readiness for Hospital Discharge Scale (RHDS) for postpartum mothers. Ginekologia polska 2019; 90(7):376–80.

30. Potkin SG, Gharabawi GM, Greenspan AJ, Rupnow MFT, Kosik-Gonzalez C, Remington G et al. Psychometric evaluation of the Readiness for Discharge Questionnaire. Schizophrenia research 2005; 80(2-3):203–12.

31. Weiss ME, Costa LL, Yakusheva O, Bobay KL. Validation of patient and nurse short forms of the readiness for hospital discharge scale and their relationship to return to the hospital. HEALTH SERV RES 2014; 49(1pt1):304–17.

32. Weiss ME, Ryan P, Lokken L. Validity and reliability of the perceived readiness for Discharge After Birth Scale. JOGNN 2006; 35(1):34–45.

33. Hodgins MJ, Filiatreault S, Keeping‚ÄêBurke L, Logan SM. Patterns of patient coping following hospital discharge from medical and surgical units: A pilot study. NURS HEALTH SCI 2020; 22(1):118–25.

34. Mixon AS, Goggins K, Bell SP, Vasilevskis EE, Nwosu S, Schildcrout JS et al. Preparedness for hospital discharge and prediction of readmission. J HOSP MED 2016; 11(9):603–9.

35. Natthawan Suwan, Sirirat Panuthai, Duangruedee Lasuka, Totsaporn Khampolsiri. Factors Influencing Readiness for Hospital Discharge Among Thai Older Persons with Chronic Obstructive Pulmonary Disease. PAC RIM INT J NURS RES 2018; 22(2):156–68.

36. Sasanuma N, Takahashi K, Itani Y, Tanaka T, Yamauchi S, Mabuchi S et al. Motor and cognitive function analysis for home discharge using the Functional Independence Measure in patients undergoing cardiac rehabilitation at a long-term acute-care hospital. European journal of physical and rehabilitation medicine 2015; 51(6):781–92.

37. Weiss ME, Yakusheva O, Bobay KL, Costa L, Hughes RG, Nuccio S et al. Effect of Implementing Discharge Readiness Assessment in Adult Medical-Surgical Units on 30-Day Return to Hospital: The READI Randomized Clinical Trial. JAMA Network Open 2019; 2(1):e187387-e187387.

38. Wong J, Wong S, Brooks E, Yabsley RH. Home readiness and recovery pattern after total hip replacement. J ORTHOP NURS 1999; 3(4):210–9.

39. Hogarty GE, Ulrich R. The discharge readiness inventory. Archives of general psychiatry 1972; 26(5):419–26.

40. Qian J, Qian M, Ren Y, Ye L, Qian F, Jin L et al. Readiness for hospital discharge and influencing factors: a cross-sectional study on patients discharged with tubes from the department of hepatobiliary surgery. BMC SURG 2021; 21(1):1–10.

41. Hu R, Gu B, Tan Q, Xiao K, Li X, Cao X et al. The effects of a transitional care program on discharge readiness, transitional care quality, health services utilization and satisfaction among Chinese kidney transplant recipients: A randomized controlled trial. INT J NURS STUD 2020; 110:103700.

42. Andrew NE, Busingye D, Lannin NA, Kilkenny MF, Cadilhac DA. The Quality of Discharge Care Planning in Acute Stroke Care: Influencing Factors and Association with Postdischarge Outcomes. Journal of stroke and cerebrovascular diseases 2018; 27(3):583–90.

43. Archer RP, Bedell JR, Amuso KF. Personality, demographic, and intellectual variables associated with readiness for discharge from psychiatric treatment. The Journal of psychology 1980; 104(1):67–74.

44. Aviram U, Minsky S, Smoyak SA, Gubman-Riesser GD. Discharge-ready patients who remain hospitalized: a re-emerging problem for mental health services. The Psychiatric quarterly 1995; 66(1):63–85.

45. Bahr SJ, Bang J, Yakusheva O, Bobay KL, Krejci J, Costa L et al. Nurse Continuity at Discharge and Return to Hospital. NURS RES 2020; 69(3):186–96.

46. Baksi A, Arda Sürücü H, I Nal G. Postcraniotomy Patients' Readiness for Discharge and Predictors of Their Readiness for Discharge. J NEUROSCI NURS 2020; 52(6):295–9.

47. Blumer V, Gayowsky A, Xie F, Greene SJ, Graham MM, Ezekowitz JA et al. Effect of patient-centered transitional care services on patient-reported outcomes in heart failure: sex-specific analysis of the PACT-HF randomized controlled trial. European journal of heart failure 2021; 23(9):1488–98.

48. Bobay KL, Conway-Phillips R, Hughes RG, Costa L, Bahr SJ, Siclovan D et al. Clinical nurses' perspectives on discharge practice changes from participating in a translational research study. Journal of Nursing Management 2021; 29(3):553–61.

49. Braet A, Weltens C, Bruyneel L, Sermeus W. The quality of transitions from hospital to home: A hospital-based cohort study of patient groups with high and low readmission rates. INT J CARE COORD 2016; 19(1/2):29–41.

50. Brent L, Coffey A. Patient’s perception of their readiness for discharge following hip fracture surgery. INT J ORTHOP TRAUMA NURS 2013; 17(4):190–8.

51. Caminsky NG, Hamad D, He BH, Zhao K, Al Mahroos M, Feldman LS et al. Optimizing discharge decision-making in colorectal surgery: a prospective cohort study of discharge practices in a recently implemented enhanced recovery pathway. Colorectal disease 2021; 23(6):1507–14.

52. Celio DA, Poggi R, Schmalzbauer M, Rosso R, Majno P, Christoforidis D. ERAS, length of stay and private insurance: a retrospective study. International journal of colorectal disease 2019; 34(11):1865–70.

53. Clark M, Steinberg M, Bischoff N. Patient readiness for return to home: discord between expectations and reality. AUST OCCUP THER J 1997; 44(3):132–41.

54. Coffey A, McCarthy GM. Older people's perception of their readiness for discharge and postdischarge use of community support and services. International journal of older people nursing 2013; 8(2):104–15.

55. Congdon JG. Managing the incongruities: the hospital discharge experience for elderly patients, their families, and nurses. APPL NURS RES 1994; 7(3):125–31.

56. Dols JD, Chargualaf KA, Gordon A, Pomerleau T, Mendoza A, Schwarzbach C et al. Relationship of Nurse-Led Education Interventions to Liver Transplant Early Readmission. PROG TRANSPLANT 2020; 30(2):88–94.

57. Du Plessis M, McGaffin CR, Molepo T, Oelofse R, van Zyl S, Mashola MK. Perceived readiness for hospital discharge: Patients with spinal cord injury versus physiotherapists. S AFR J PHYSIOTHER 2018; 74(1):437.

58. Rotvig C, Christensen AV, Rasmussen TB, Borregaard B, Thrysoee L, Juel K et al. Unreadiness for hospital discharge predicts readmission among cardiac patients: results from the national DenHeart survey. European journal of cardiovascular nursing 2021.

59. Wallace AS, Pierce NL, Davisson E, Manges K, Tripp-Reimer T. Social resource assessment: Application of a novel communication tool during hospital discharge. PATIENT EDUC COUNS 2018; 102(3):542–9.

60. Grimmer KA, Dryden LR, Puntumetakul R, Young AF, Guerin M, Deenadayalan Y et al. Incorporating patient concerns into discharge plans: evaluation of a patient-generated checklist. INTERNET J ALLIED HEALTH SCI PRACT 2006; 4(2):1–23.

61. Handy IA. Judgments of release readiness as related to discharge from a neuropsychiatric hospital. COMMUNITY MENT HEALTH J 1974; 10:198–204.

62. Harrison JD, Boscardin WJ, Maselli J, Auerbach AD. Does Feedback to Physicians of a Patient-Reported Readiness for Discharge Checklist Improve Discharge? Journal of patient experience 2020; 7(6):1144–50.

63. Harrison JD, Greysen RS, Jacolbia R, Nguyen A, Auerbach AD. Not ready, not set_discharge: Patient-reported barriers to discharge readiness at an academic medical center. J HOSP MED 2016; 11(9):610–4.

64. Heine J, Koch S, Goldie P. Patients' experiences of readiness for discharge following a total hip replacement. AUST J PHYSIOTHER 2004; 50(4):227–33.

65. Hogarty GE. Hospital differences in the release of discharge ready chronic schizophrenics. Archives of general psychiatry 1968; 18(3):367–72.

66. Hydzik P, Kolarczyk E, Kustrzycki W, Kubielas G, Ka_u_na-Oleksy M, Szczepanowski R et al. Readiness for Discharge from Hospital after Myocardial Infarction: A Cross-Sectional Study. International journal of environmental research and public health 2021; 18(13).

67. Jack BW, Chetty VK, Anthony D, Greenwald JL, Sanchez GM, Johnson AE et al. A reengineered hospital discharge program to decrease rehospitalization: a randomized trial. ANN INTERN MED 2009; 150(3):178–87.

68. Yang J, Yuhua He, Lili Jiang, Ka Li, Yang J, He Y et al. Colorectal patients' readiness for hospital discharge following management of enhanced recovery after surgery pathway: A cross-sectional study from China. MEDICINE 2020; 99(8):1–6.

69. Kaya S, SAIN GUVEN G, AYDAN S, Kar A, Teleş M, Yıldız A et al. Patients' readiness for discharge: Predictors and effects on unplanned readmissions, emergency department visits and death. Journal of Nursing Management 2018; 26(6):707–16.

70. Kelly A, Watson D, Raboud J, Bilsker D. Factors in delays in discharge from acute-care psychiatry. Canadian journal of psychiatry. Revue canadienne de psychiatrie 1998; 43(5):496–501.

71. Kleinpell RM. Randomized trial of an intensive care unit-based early discharge planning intervention for critically ill elderly patients. AM J CRIT CARE 2004; 13(4):335–45.

72. Kosobucka A, Michalski P, Pietrzykowski Ł, Kasprzak M, Fabiszak T, Felsmann M et al. The impact of readiness to discharge from hospital on adherence to treatment in patients after myocardial infarction. Cardiol J. 2022;29(4):582-590.

73. LaManna JB, Bushy A, Norris AE, Chase SK. Early and Intermediate Hospital-to-Home Transition Outcomes of Older Adults Diagnosed With Diabetes. DIABETES EDUC 2016; 42(1):72–86.

74. Lau D, Padwal RS, Majumdar SR, Pederson JL, Belga S, Kahlon S et al. Patient-Reported Discharge Readiness and 30-Day Risk of Readmission or Death: A Prospective Cohort Study. AM J MED 2016; 129(1):89–95.

75. Liang W, Zuo D, Candidate M, Li T, Zhao H. Patient-Readiness for Discharge and 30-Day Adherence to Treatment After Coronary Stent Implantation. CLIN NURS RES 2021; 30(8):1271–80.

76. Mabire C, Büla C, Morin D, Goulet C. Nursing discharge planning for older medical inpatients in Switzerland: A cross-sectional study. GERIATR NURS 2015; 36(6):451–7.

77. Mabire C, Bachnick S, Ausserhofer D, Simon M. Patient readiness for hospital discharge and its relationship to discharge preparation and structural factors: A cross-sectional study. INT J NURS STUD 2019; 90:13–20.

78. Malagon-Maldonado G, Connelly CD, Bush RA. Predictors of Readiness for Hospital Discharge After Birth: Building Evidence for Practice. WORLDVIEWS EVID BASED NURS 2017; 14(2):118–27.

79. Manges KA, Wallace AS, Groves PS, Schapira MM, Burke RE. Ready to Go Home? Assessment of Shared Mental Models of the Patient and Discharging Team Regarding Readiness for Hospital Discharge. J HOSP MED 2020; 16(6):326–32.

80. Meng N, Liu R, Wong M, Liao J, Feng C, Li X. The association between patient-reported readiness for hospital discharge and outcomes in patients diagnosed with anxiety disorders: A prospective and observational study. Journal of Psychiatric & Mental Health Nursing 2020; 27(4):380–92.

81. Meo N, Bann M, Sanchez M, Reddy A, Cornia PB. Getting Unstuck: Challenges and Opportunities in Caring for Patients Experiencing Prolonged Hospitalization While Stable for Discharge. AM J MED 2020; 133(12):1406–10.

82. Mess E, Ziembowska A, Sta_ J, Misi_g W, Chabowski M. Assessment of the emotional condition of women with cancer. European review for medical and pharmacological sciences 2021; 25(17):5429–35.

83. Middleton S, Appleberg M, Girgis S, Ward JE. Effective discharge policy: are we getting there? AUST HEALTH REV 2004; 28(3):255–9.

84. Nurhayati N, Songwathana P, Vachprasit R. Surgical patients' experiences of readiness for hospital discharge and perceived quality of discharge teaching in acute care hospitals. J CLIN NURS 2019; 28(9/10):1728–36.

85. Opper K, Beiler J, Yakusheva O, Weiss M. Effects of Implementing a Health Team Communication Redesign on Hospital Readmissions Within 30 Days. WORLDVIEWS EVID BASED NURS 2019; 16(2):121–30.

86. Peyrovi H, Mosayebi Z, Mohammad-Doost F, Chehrzad M-M, Mehran A. The effect of empowerment program on "perceived readiness for discharge" of mothers of premature infants. J MATERN FETAL NEONAT MED 2016; 29(5):752–7.

87. Presciutti A, Shaffer JA, Newman M, Perman SM. Modifiable provider-patient relationship factors and illness perceptions are associated with quality of life in survivors of cardiac arrest with good neurologic recovery. Resuscitation plus 2020; 3:100008

88. Rodrigue N, Laizner AM, Tze N, Sewitch M. Experiences of Older Adult Trauma Patients Discharged Home From a Level I Trauma Center. J TRAUMA NURS 2017; 24(3):182–92.

89. Saunders S, Weiss ME, Meaney C, Killackey T, Varenbut J, Lovrics E et al. Examining the course of transitions from hospital to home-based palliative care: A mixed methods study. PALLIAT MED 2021; 35(8):1590–601.

90. Schaefer AL, Anderson JE, Simms LM. Are they ready? Discharge planning for older surgical patients. Journal of gerontological nursing 1990; 16(10):16–9.

91. Schmocker RK, Holden SE, Vang X, Leverson GE, Cherney Stafford LM, Winslow ER. Association of Patient-Reported Readiness for Discharge and Hospital Consumer Assessment of Health Care Providers and Systems Patient Satisfaction Scores: A Retrospective Analysis. J AM COLL SURG 2015; 221(6):1073-1082.e3.

92. Schneider MA, Howard KA. Using Technology to Enhance Discharge Teaching and Improve Coping for Patients After Stroke. J NEUROSCI NURS 2017; 49(3):152–6.

93. Siow E, Lo SM, Yeung KL, Yeung RSD, Choi KC, Chair SY. Factors and post-discharge outcomes associated with patients' readiness for discharge from the emergency medicine ward: A prospective study. INT EMERG NURS 2019; 46:100773.

94. Sriprasong S, Hanucharurnkul S, Panpukdee O, Krittayaphong R, Pongthavornkamol K, Vorapongsathorn T. Functional status model: an empirical test among discharged acute myocardial infarction patients. THAI J NURS RES 2009; 13(4):268–84.

95. Verhaegh KJ, Jepma P, Geerlings SE, Rooij SE, Buurman BM. Not feeling ready to go home: a qualitative analysis of chronically ill patients' perceptions on care transitions. INT J QUAL HEALTH CARE 2019; 31(2):125–32.

96. Wallace AS, Perkhounkova Y, Bohr NL. Quality of Transition From Hospital to Home: The Influence of Nurse- and Patient-Reported Readiness. CLIN NURS RES 2018; 27(2):129–47.

97. Wallace AS, Perkhounkova Y, Bohr NL, Chung SJ. Readiness for Hospital Discharge, Health Literacy, and Social Living Status. CLIN NURS RES 2016; 25(5):494–511.

98. Wang M, Lv L, Yu Z, Gao L, Lu Q, Ou J et al. A cross-sectional study of readiness for discharge, chronic illness resources and postdischarge outcomes in patients with diabetic foot ulcer. Nursing open 2021; 8(5):2645–54.

99. Wang M, Wang Y, Meng N, Li X. The factors of patient-reported readiness for hospital discharge in patients with depression: A cross-sectional study. Journal of Psychiatric & Mental Health Nursing 2021; 28(3):409–21.

100. Weiss M, Ryan P, Lokken L, Nelson M. Length of stay after vaginal birth: sociodemographic and readiness-for-discharge factors. BIRTH 2004; 31(2):93–101.

101. Weiss ME, Lokken L. Predictors and outcomes of postpartum mothers' perceptions of readiness for discharge after birth. JOGNN 2009; 38(4):406–17.

102. Weiss ME, Piacentine LB, Lokken L, Ancona J, Archer J, Gresser S et al. Perceived readiness for hospital discharge in adult medical-surgical patients. CLIN NURSE SPEC 2007; 21(1):31–42.

103. Weiss ME, Yakusheva O, Bobay KL. Quality and cost analysis of nurse staffing, discharge preparation, and postdischarge utilization. HEALTH SERV RES 2011; 46(5):1473–94.

104. Xiong L, Liu Y, Chen Q, Tian Y, Yang M. Readiness for Hospital Discharge of Adult Patients with Major Depressive Disorder in China: A Cross-Sectional Study. Patient preference and adherence 2021; 15:1681–92.

105. Yanıkkerem E, Esmeray N, Karakuş A, Üstgörül S, Baydar Ö, Göker A. Factors affecting readiness for discharge and perceived social support after childbirth. J CLIN NURS 2018; 27(13-14):2763–75.

106. Zhang A, Feng X, Qiu C, Violeta L. Discharge teaching, readiness for hospital discharge and post-discharge outcomes in cataract patients: A structural equation model analysis. Journal of Nursing Management 2021; 29(3):543–52.

107. Zhao H, Feng X, Yu R, Gu D, Zhang X. Factors influencing readiness for hospital discharge among patients undergoing laryngectomy. International Journal of Nursing Practice (John Wiley & Sons, Inc.) 2020; 26(5):1–8.
